# Synthesis, electronic structures, and photoluminescence properties of an efficient and thermally stable red-emitting phosphor Ca_3_ZrSi_2_O_9_:Eu^3+^,Bi^3+^ for deep UV-LEDs

**DOI:** 10.1039/c8ra00844b

**Published:** 2018-04-09

**Authors:** Jiyou Zhong, Weiren Zhao, Lunwei Yang, Peng Shi, Zifeng Liao, Menglong Xia, Wenhua Pu, Wei Xiao, Ligen Wang

**Affiliations:** School of Physics and Optoelectronic Engineering, Guangdong University of Technology Guangzhou 510006 China zwren123@126.com; Center for Computing and Simulation of Advanced Materials, State Key Laboratory of Nonferrous Metals and Processes, General Research Institute for Nonferrous Metals Beijing 100088 China lg_wang1@yahoo.com

## Abstract

A series of red-emitting Ca_3_ZrSi_2_O_9_:Eu^3+^,*x*Bi^3+^ phosphors was synthesized using a conventional high temperature solid-state reaction method, for the purpose of promoting the emission efficiency of Eu^3+^ in a Ca_3_ZrSi_2_O_9_ host. The site preference of Bi^3+^ and Eu^3+^ in the Ca_3_ZrSi_2_O_9_ host was evaluated by formation energy. The effects of Bi^3+^ on electronic structure, luminescent properties, and related mechanisms were investigated. The inner quantum yield of the optimized sample increased to 72.9% (*x* = 0.08) from 34.6% (*x* = 0) at 300 nm ultraviolet light excitation. The optimized sample (*x* = 0.08) also showed excellent thermal stability, and typically, 84.2% of the initial emission intensity was maintained when the temperature increased to 150 °C from 25 °C, which is much higher than that without Bi^3+^ doping (70.1%). The mechanisms of emission properties and thermal stability enhancement, as well as the redshift of the charge transfer band (CTB) induced by Bi^3+^ doping in the Ca_3_ZrSi_2_O_9_:Eu^3+^ phosphor, were discussed. This study elucidates the photoluminescence properties of Bi^3+^-doped Ca_3_ZrSi_2_O_9_:Eu^3+^ phosphor, and indicates that it is a promising luminescent material that can be used in ultraviolet light-emitting diodes.

## Abstract 2

A red-emitting phosphor Ca_3_ZrSi_2_O_9_:Eu^3+^,Bi^3+^ with high quantum yield and thermal stability was developed by introducing Bi^3+^ as an efficient sensitizer.

## Graphical abstract

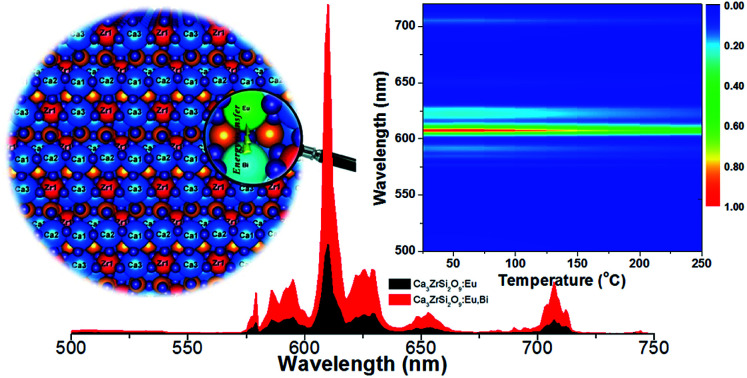

## 1. Introduction

Phosphor-converted white light-emitting diodes (pc-WLEDs) have the advantages of long lifetime, high luminous efficacy, and environmental friendliness, and are becoming an indispensable solid-state light source in our daily life.^1–5^ However, conventional WLEDs using blue InGaN chips combined with phosphors are very likely to cause health problems because excessive exposure to monochromatic blue light often results from their use.^6,7^ Therefore, ultraviolet (UV) LEDs combined with phosphors are an important and promising alternative for the fabrication of full spectrum lighting sources that are healthy and comfortable to human beings.^8,9^ With their rapid development, UV LEDs are mainly divided into near-UV LEDs (300–400 nm) and deep UV LEDs (200–300 nm).^10^ Correspondingly, new highly efficient and thermally stable phosphors should be rapidly developed so as to match the emission wavelength of UV LED chips.^11^ Generally, Y_2_O_3_:Eu^3+^ is considered a red-emitting phosphor for UV LEDs that are solely composed of rare earth elements. Because rare earth elements are non-renewable resources, it is necessary to develop efficient and thermally stable red-emitting phosphors with inexpensive raw materials and comparative properties to replace the use of Y_2_O_3_:Eu^3+^ in UV LEDs.

Recently, Kim *et al.* reported a cuspidine-type Ca_3_ZrSi_2_O_9_:Eu^3+^ red-emitting phosphor possessing a relative emission intensity that reached 84% of commercial red-emitting Y_1.94_Eu_0.06_O_3_ phosphors, and it seemed promising as a replacement for Y_2_O_3_:Eu^3+^, but the inner quantum yield was only 41% at 268 nm excitation.^12^ In order to further enhance the emission efficiency, Zuo *et al.* tried to partially substitute Zr^4+^ ions with Al^3+^ ions in the host Ca_3_ZrSi_2_O_9_, and finally the inner quantum yield was increased to 46%.^13^ This enhancement was mainly ascribed to the charge compensation effect, which is helpful for improving the crystallinity of the phosphors. However, the relatively low quantum yield still limits its practical use.

Here, we report another route for enhancing the quantum yield of this phosphor by introducing the sensitizer Bi^3+^ into the lattice. It is known that Bi^3+^ has an ns^2^-type ground state electric configuration (^1^S_0_ state) and ^1^S_0_ → ^3^P_1_ and ^1^S_0_ → ^1^P_1_ spin-allowed transitions, which are expected to have a reasonable absorption strength.^14^ The aborted energy can be efficiently transferred to luminescence center ions, such as Eu^3+^ and Mn^4+^, which act as a sensitizer, or directly emitting photons, acting as a luminescence center due to the transitions of ^3^P_1_ → ^1^S_0_ and ^1^P_1_ → ^1^S_0_.^15–19^ For this reason, many commercially available Eu^3+^-activated phosphors, such as Y_2_O_3_:Eu^3+^ and Y(P,V)O_4_:Eu^3+^, adopt Bi^3+^ as sensitizer.^20–22^ In this approach, the inner quantum yield of Ca_3_ZrSi_2_O_9_:Eu^3+^,Bi^3+^ was greatly promoted, and the thermal stability was also enhanced. To further understand this enhancement, first principles calculations were performed to investigate the site preference of dopants and electronic structure variations induced by Bi^3+^ doping. The synthesis and photoluminescence properties were also experimentally characterized in detail. The photoluminescence investigation demonstrates that the Ca_3_ZrSi_2_O_9_:Eu^3+^,Bi^3+^ phosphor is a promising red-emitting phosphor that can be incorporated into UV LEDs.

## 2. Methodology

### 2.1 Experimental details

The samples with formula (Ca_2.83−*x*_Eu_0.17_Bi_*x*_)ZrSi_2_O_9_ (*x* = 0–0.16) were synthesized by the high-temperature solid-state reaction method. The starting materials consisting of CaCO_3_ (Aldrich, 99.95%), Bi_2_O_3_ (Aldrich, 99.9%), ZrO_2_ (Aldrich, 99.5%), SiO_2_ (Aldrich, 99.99%) and Eu_2_O_3_ (Aldrich, 99.95%) were weighed according to the stoichiometric ratio. The mixed powder was evenly ground in an agate mortar, and then the homogeneous mixtures were placed in an alumina crucible and continually heated at 1400 °C in an air atmosphere for 6 h. The samples were gradually cooled to room temperature and then ground once more until a fine powder was obtained.

The powder X-ray diffraction (XRD) patterns were measured using an X-ray powder diffractometer (Rigaku, Japan) with Cu-Kα radiation (*λ* = 1.5406 Å). The photoluminescence spectra and the decay curves of Eu^3+^ lifetime values were measured using a FLS-980 fluorescence spectrophotometer (Edinburgh Instruments) equipped with a xenon lamp (450 W, Osram) as the excitation source. The quantum yield and temperature-dependent emission spectra were measured using the QE-2100 quantum yield measurement system (Otsuka Electronics Co., Ltd., Japan), composed of an integrating sphere, a heating apparatus, and a Xe lamp used as an excitation source and white BaSO_4_ powder as a reference.

### 2.2 Computational details

Structural optimization of Ca_3_ZrSi_2_O_9_, Ca_3_ZrSi_2_O_9_:Bi^3+^, and Ca_3_ZrSi_2_O_9_:Eu^3+^ was firstly performed using the density functional theory (DFT) method with the Perdew–Burke–Ernzerhof (PBE) exchange-correlation functional,^23^ as implemented in the VASP software package.^24,25^ The electronic properties of Ca_3_ZrSi_2_O_9_:Bi^3+^ were then calculated based on the optimized geometries using the hybrid PBE functional. The Ca_3_ZrSi_2_O_9_ host crystal containing 60 atoms was used as the computational model. The Ca 3s^2^3p^6^4s^2^, Zr 4s^2^4p^6^5s^2^4d^2^, Si 3s^2^3p^2^, O 2s^2^2p^4^, Bi 5d^10^6s^2^6p^3^, and Eu 5s^2^5p^6^4f^6^5d^1^6s^2^ electrons were treated as the valence electrons, whose interactions with the ion cores were treated with the projected augmented wave (PAW) method.^26^ The geometric structures were fully relaxed with the convergence criteria of 10^−6^ eV used for the change in the total energy and 0.01 eV Å^−1^ used for Hellmann–Feynman forces on atoms. The cut-off energy of 550 eV was used for the basis set of the plane waves. The Brillouin zone integrations were sampled using a 4 × 2 × 2 Monkhorst–Pack *k-*point mesh.

## 3. Results and discussion

### 3.1 Phase purity, crystal structure, and site preference of dopants

**Fig. 1:**
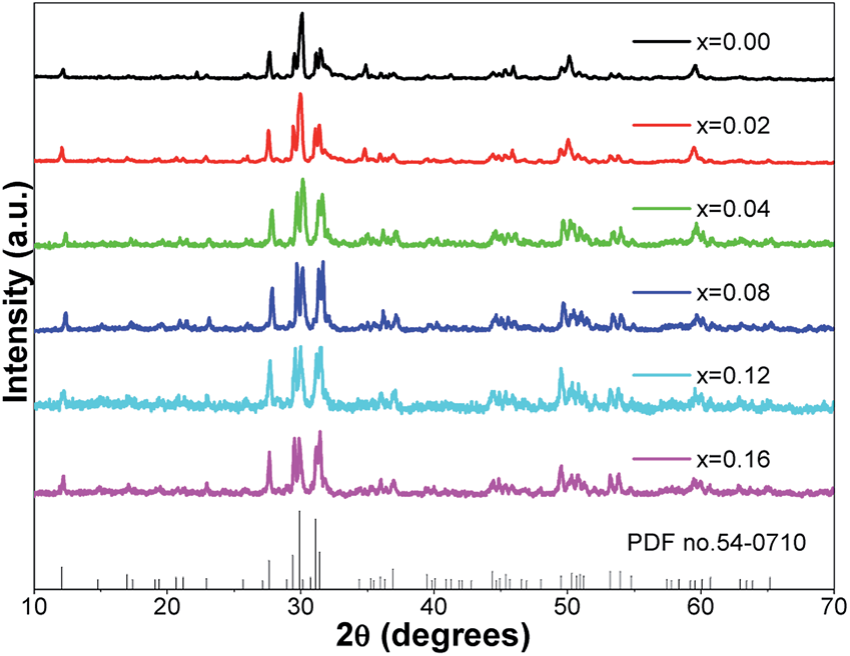
Powder XRD patterns of the synthesized (Ca_2.83−*x*_Eu_0.17_Bi_*x*_)ZrSi_2_O_9_ (*x* = 0–0.16) samples, and the standard card (PDF no. 54-0710) of Ca_3_ZrSi_2_O_9_ is given for comparison.

**Fig. 2:**
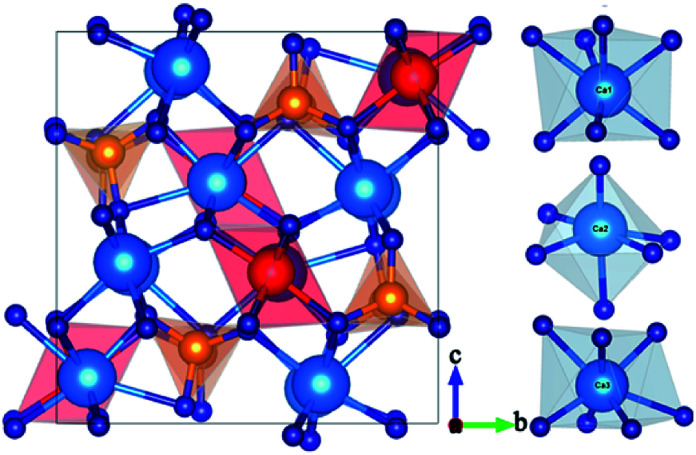
Crystal structure of Ca_3_ZrSi_2_O_9_ views along the (100) direction; and three types of Ca sites, namely, Ca1, Ca2, and Ca3 with seven-, six-, and seven-coordinations, respectively.

Fig. 1 presents the powder XRD patterns for the synthesized (Ca_2.83−*x*_Eu_0.17_Bi_*x*_)ZrSi_2_O_9_ (*x* = 0–0.16) samples compared with the standard pattern (PDF no. 54-0710) for Ca_3_ZrSi_2_O_9_. All the diffraction peaks of these samples can be indexed to the *P*2_1_/*c* space group (no. 14) of the monoclinic system,^27^ indicating that a single phase with high purity has been synthesized, and the doped Bi^3+^ and Eu^3+^ did not generate any impurity or induce significant changes in the host structure. The Ca_3_ZrSi_2_O_9_ compound can be regarded as being derived from the well-known cuspidine Ca_4_Si_2_O_7_F_2_ by substituting Zr-O_2_ for Ca-F_2_.^28^ Thus, the crystal structure (shown in Fig. 2) of Ca_3_ZrSi_2_O_9_ is similar to Ca_4_Si_2_O_7_F_2_, composed of [SiO_4_] tetrahedrons, [ZrO_6_] octahedrons, [CaO_6_] octahedrons, and [CaO_7_] decahedrons, forming a three dimensional network. There are three types of Ca^2+^ sites and one type of Zr^4+^ site, and they are available for Eu^3+^ and Bi^3+^ to occupy.

To identify the site preference, the formation energy *E*_form_ was calculated according to the following equation:^29,30^

**Fig. 3:**
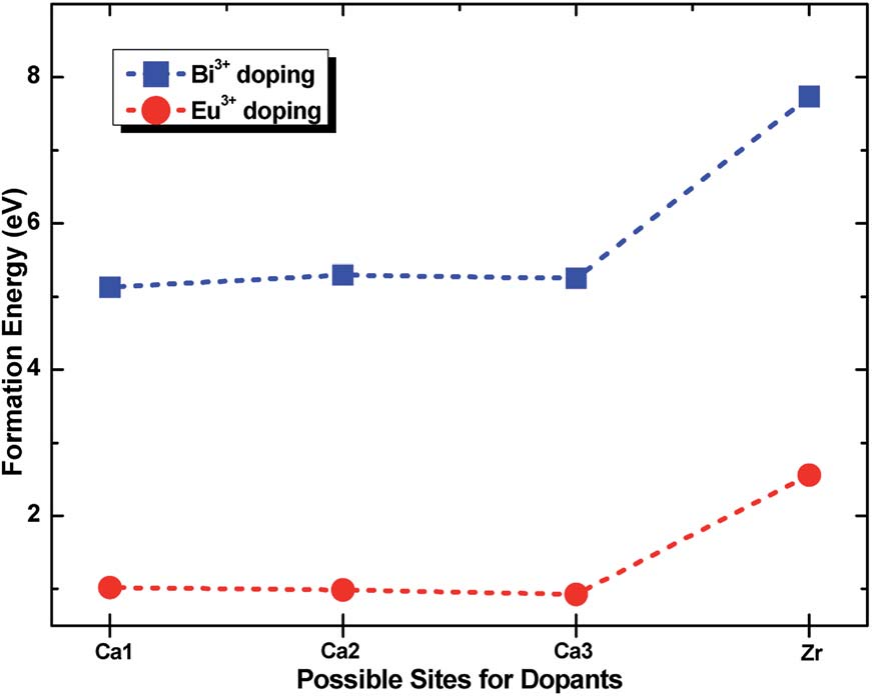
Formation energy of Eu^3+^ and Bi^3+^ doping into Zr^4+^ and three types of Ca^2+^ sites in the Ca_3_ZrSi_2_O_9_ host.

1 *E*_form_ = *E*_M:CZSO_ − *E*_CZSO_ + *μ*_Ca or Zr_ − *μ*_M_

where CZSO refers to Ca_3_ZrSi_2_O_9_, M represents metal Eu or Bi, *E*_M:CZSO_ and *E*_CZSO_ is the total energy of the metal-doped CZSO system and undoped CZSO, respectively, and *μ* is the total energy per atom of the bulk metal. According to this equation, the formation energies of Eu^3+^ and Bi^3+^ at possible cation sites were calculated and are shown in Fig. 3. As presented, both Eu^3+^ and Bi^3+^ exhibit much lower formation energy in the Ca^2+^ sites than in the Zr^4+^ site. The formation energy for Eu^3+^ and Bi^3+^ in three types of Ca^2+^ sites is very similar, but Eu^3+^ entering Ca_(3)_ and Bi^3+^ entering Ca_(1)_ provide slightly lower formation energy. These results indicate that Eu^3+^ and Bi^3+^ only occupy Ca^2+^ sites, but nearly have equal possibility to enter any type of Ca^2+^ site during the high temperature sintering process. The formation energy results also suggest that it is much more difficult to dope Bi^3+^ into the lattice than Eu^3+^, mainly due to its larger ionic radius and size mismatch effect.

### 3.2 Electronic structure of Bi^3+^-doped and undoped Ca_3_ZrSi_2_O_9_

**Fig. 4:**
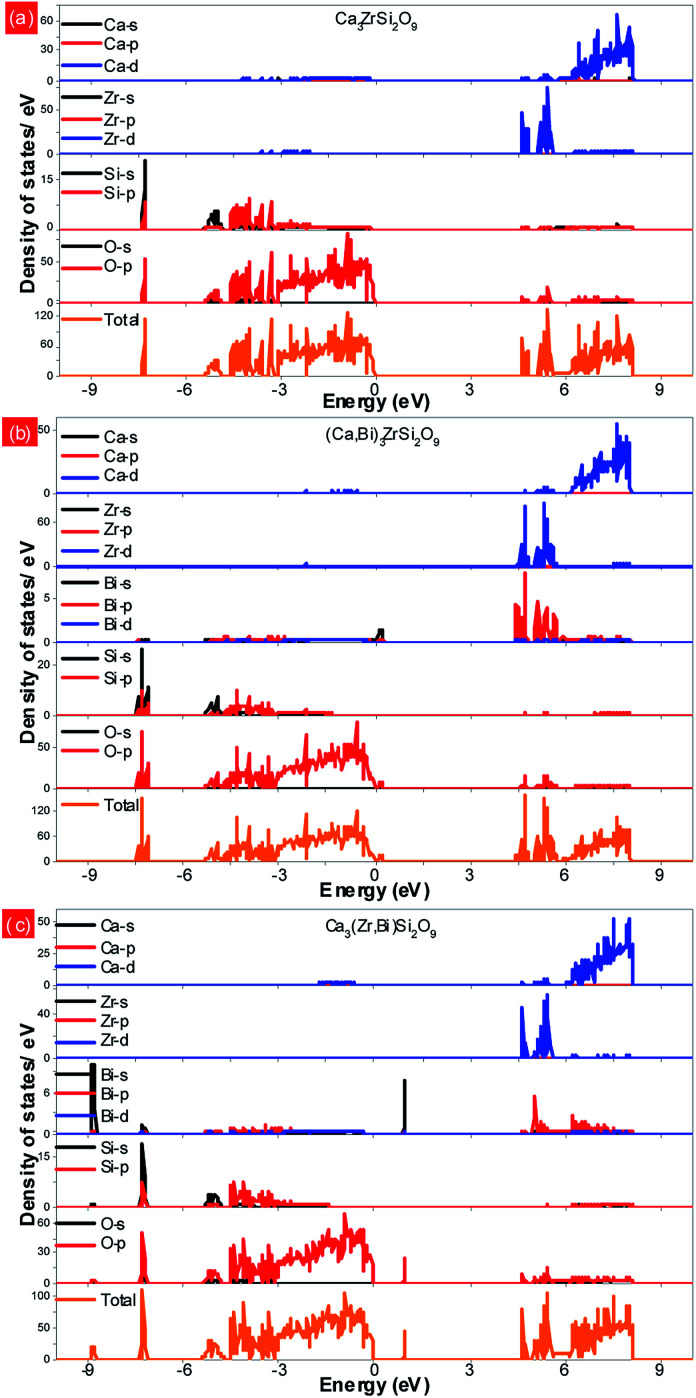
Total and partial density of states of the (a) Ca_3_ZrSi_2_O_9_ host; (b) Bi^3+^ doping into the Ca_(1)_ site; and (c) Bi^3+^ doping into the Zr^4+^ site.

Fig. 4a presents the total and partial density of states (DOSs) of the Ca_3_ZrSi_2_O_9_ host. As shown, the conduction band is mainly composed of Ca 3d and Zr 4d states, which show a hybridization character with O 2p and Si 3p states. The bottom of the conduction band is mainly determined by Zr 4d states. The top of the valence band reflects the p electronic character, deriving from O 2p states. The valence bands at the lower energy are mainly attributed to Si 3s/3p and O 2s/2p states. The band gap is calculated to be 4.59 eV, corresponding to the optical absorption at 270 nm of Zr^4+^–O^2−^. This energy value is slightly underestimated compared to the experimentally observed value of 255 nm,^31^ due to the drawback of the DFT method.^32^

Fig. 4b shows the total and partial density of states of Bi doped in the most possible Ca_(1)_ site. In this case, the bottom of the conduction band is mainly composed of Zr 4d and Bi 6p states, and a strong hybridization between them can be observed. The p electronic character of the O 2p states remains at the top of the valence band. Additionally, the Bi 6s states emerge at the band gap, slightly above the top of the valence band (by approximately 0.18 eV), presenting a strong hybridization with O 2p states. The band gap is reduced to 4.41 eV.

The total and partial density of states of Bi doped in the Zr site were also investigated and are presented in Fig. 4c. In this situation, the Bi 6p states are higher than the Zr 4d states, and thus, the bottom of the conduction band is determined by the Zr 4d states. The top of the valence band still presents the p electronic character of O 2p states, while the location of Bi 6s is significantly higher than that with Bi doping at the Ca_(1)_ site. Also, a strong hybridization between the Bi 6s and O 2p states can be detected. The band gap is not influenced by the doped Bi and remained at 4.59 eV.

### 3.3 Photoluminescence properties of Ca_3_ZrSi_2_O_9_:Eu^3+^,*x*Bi^3+^

**Fig. 5:**
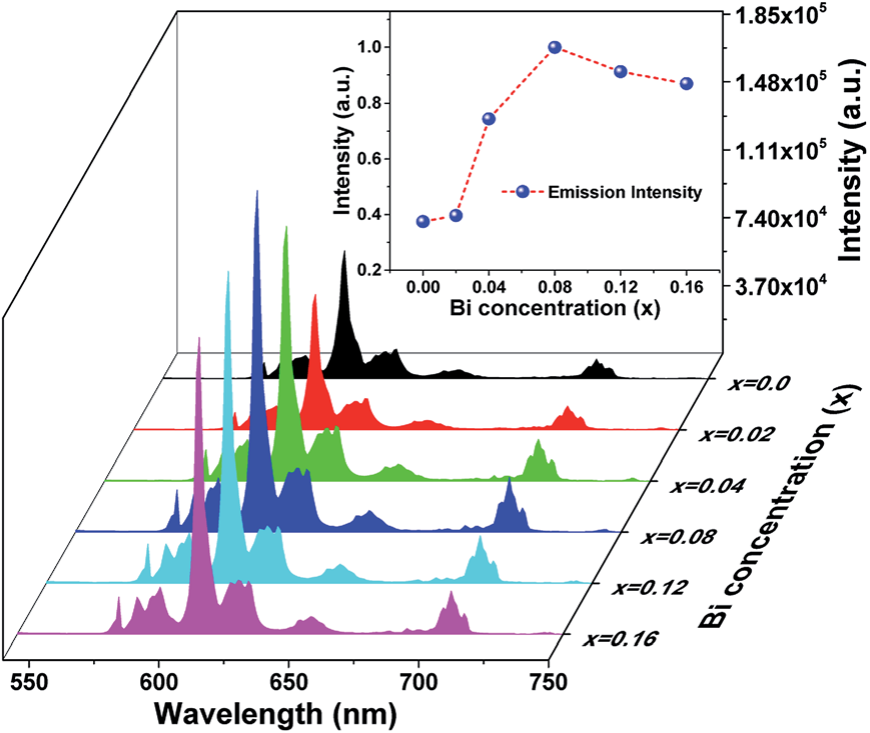
The photoluminescence emission spectra of samples (Ca_2.83−*x*_Eu_0.17_Bi_*x*_)ZrSi_2_O_9_ (*x* = 0–0.16) excited by 300 nm UV light. The emission intensity of the dominant peak (610 nm) as a function of Bi^3+^ concentration was inserted.

The photoluminescence emission (PL) spectra of samples (Ca_2.83−*x*_Eu_0.17_Bi_*x*_)ZrSi_2_O_9_ (*x* = 0–0.16) excited by 300 nm UV light are presented in Fig. 5. As shown, the spectrum exhibits typical characteristics of Eu^3+^, with several sharp emission peaks located at 579 nm, 586/595 nm, 610/622/626/630 nm, 648/654 nm, and 704/707/709/712 nm corresponding to the transitions of ^5^D_0_ → ^7^F_*J*_ (*J* = 0–4),^33,34^ respectively. The emission intensity gradually increases and then decreases with the increasing concentration of Bi^3+^ doping. The optimized Bi^3+^ concentration is *x* = 0.08, with an inner quantum yield of 72.9% and external quantum yield of 59.7% under 300 nm excitation, while the sample without Bi^3+^ doping has an inner quantum yield of 34.6% and external quantum yield of 27.3% under the same measured conditions. This emission enhancement is mainly ascribed to the sensitization provided by Bi^3+^ because Bi^3+^ can absorb energy through ^1^S_0_ → ^3^P_1_ and ^1^S_0_ → ^1^P_1_ transitions and transfer its energy to Eu^3+^, improving the absorbance of this phosphor. However, when the Bi^3+^ doping concentration exceeds *x* = 0.08, more defects may form due to nonequivalence substitution, resulting in a slight decrease in emission intensity.^35^

**Fig. 6:**
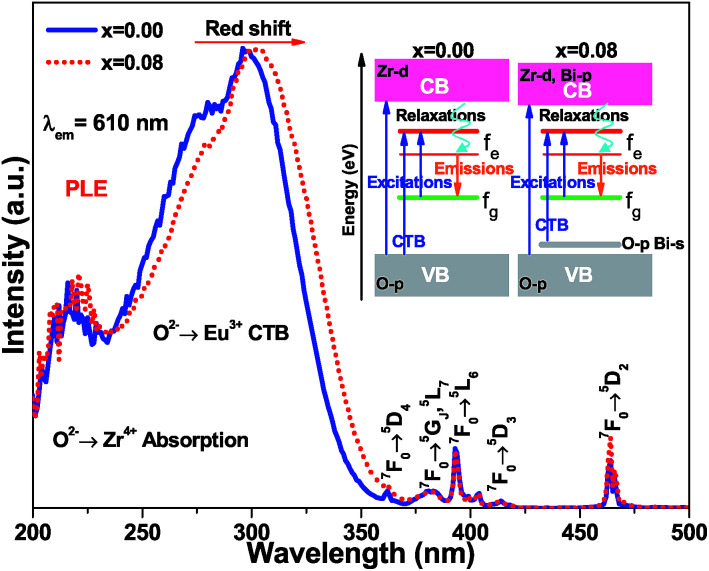
The normalized photoluminescence excitation spectra of (Ca_2.83−*x*_Eu_0.17_Bi_*x*_)ZrSi_2_O_9_ (*x* = 0 and 0.08) monitored at 610 nm. A sketch of involved energy level variations was inserted.

The normalized photoluminescence excitation spectra of (Ca_2.83−*x*_Eu_0.17_Bi_*x*_)ZrSi_2_O_9_ (*x* = 0 and 0.08) monitored at 610 nm are compared and shown in Fig. 6. It can be observed that the excitation peaks ascribing to f–f transitions of Eu^3+^ are in good agreement at each wavelength, while the charge transfer band (CTB) presents a redshift of approximately 10 nm (approximately 0.14 eV) when Bi^3+^ was introduced in this phosphor, which indicates that the 4f energy levels are not influenced by Bi^3+^ doping, but the band gap and Eu^3+^–O^2−^ interactions may have been affected.^16,36^ This redshift is mainly ascribed to the existence of a hybrid level constituted by O 2p and Bi 6s states above the valence band, which decrease the energy between the O 2p and Eu 4f states. The energy difference (approximately 0.14 eV) corresponding to the redshift in CTB is very close to the energy separation (approximately 0.18 eV) between the above mentioned hybrid level and the top of the valence band obtained by DFT calculation (shown in Fig. 4b), which further confirms that Bi^3+^ prefers to enter Ca^2+^ sites, especially the Ca_(1)_ site. Additionally, we can also find the redshift of the Zr^4+^–O^2−^ absorption band at a high energy, and this shift should be ascribed to the lowering of the conduction band induced by Bi^3+^ doping. The involved energy level variations are shown in the sketch inserted in Fig. 6.

**Fig. 7:**
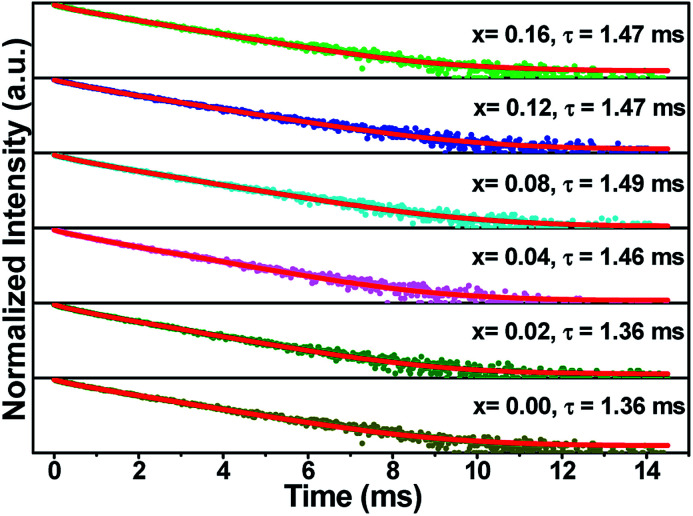
The normalized fluorescence decay curves of (Ca_2.83−*x*_Eu_0.17_Bi_*x*_)ZrSi_2_O_9_ (*x* = 0–0.16) excited by 300 nm UV light and monitored at 610 nm, and lifetimes that were calculated by fitting two exponential functions.

Fig. 7 illustrates the normalized fluorescence decay curves of (Ca_2.83−*x*_Eu_0.17_Bi_*x*_)ZrSi_2_O_9_ (*x* = 0–0.16) excited by 300 nm UV light and monitored at 610 nm. These decay curves are unable to be fitted by a single exponential function, but can be well fitted by a two-exponential function. Generally, Eu^3+^ occupies three types of Ca^2+^ sites, and it is more reasonable to fit the decay curves by a three-exponential function than a two-exponential function. However, due to the similarity between the coordination numbers and symmetry of the Ca_(1)_ site and the Ca_(3)_ site, Eu^3+^ enters either the Ca_(1)_ site or Ca_(3)_ site, and the luminescent properties are quite similar. Thus, it is reasonable to fit the decay curves by a two-exponential function. In this method, the decay times were determined to be 1.36 ms, 1.36 ms, 1.46 ms, 1.49 ms, 1.47 ms, and 1.47 ms for (Ca_2.83−*x*_Eu_0.17_Bi_*x*_)ZrSi_2_O_9_ (*x* = 0–0.16) phosphors with increasing Bi^3+^ concentration. In terms of the values of decay times, it is difficult to find obvious regularity, but a trend is exhibited of first an increase and then a decrease. The reason for the increased decay time is mainly due to the energy transfer from Bi^3+^ to Eu^3+^, while a further increase in Bi^3+^ concentration will create more defects that trap the excited electrons, reducing the decay times.^37,38^

**Fig. 8:**
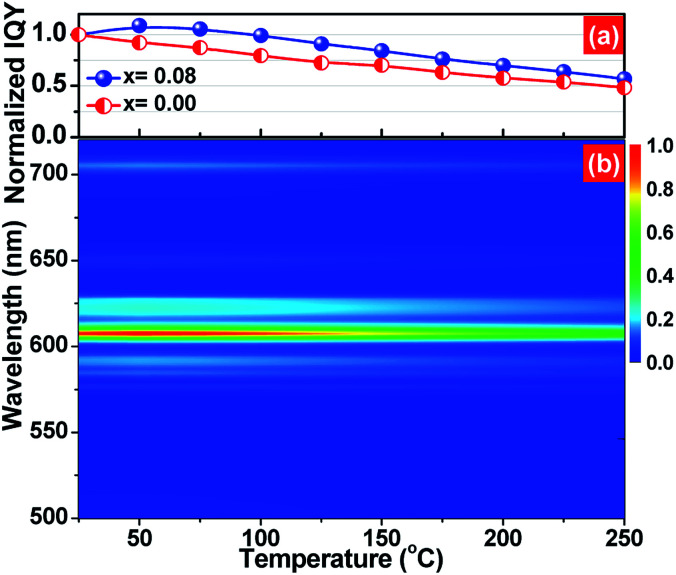
(a) The temperature-dependent normalized inner quantum yield of (Ca_2.83−*x*_Eu_0.17_Bi_*x*_)ZrSi_2_O_9_ (*x* = 0 and 0.08) phosphors under 300 nm excitation and (b) the temperature-dependent emission spectrum of the (Ca_2.83−*x*_Eu_0.17_Bi_*x*_)ZrSi_2_O_9_ (*x* = 0.08) phosphor.

The thermal quenching property, namely emission loss with increasing temperature, is one of the key application criteria for practical phosphors.^39^ The temperature-dependent normalized inner quantum yield of (Ca_2.83−*x*_Eu_0.17_Bi_*x*_)ZrSi_2_O_9_ (*x* = 0 and 0.08) phosphors under 300 nm UV excitation are presented in Fig. 8a. It can be detected that the Bi^3+^-doped phosphor has better thermal stability than that without Bi^3+^ doping at any point in the measured temperature range (25–250 °C). We observed that the quantum yield as well as emission intensity (shown as Fig. 8b) first increases up to a temperature of 50 °C and then decreases in the (Ca_2.83−*x*_Eu_0.17_Bi_*x*_)ZrSi_2_O_9_ (*x* = 0.08) phosphor. The photoluminescence (PL) intensity increase is generally ascribed to the formation of defect levels in the phosphor, which can capture electrons and then release them with increasing temperature, causing the increase of the PL intensity.^40,41^ Because of the effect of defects in increasing the thermal stability, this Bi^3+^-doped phosphor can remain at 84.2% of the initial quantum yield (at room temperature) when the temperature was increased up to 150 °C. The yield is better than that of Y_2_O_3_:Eu^3+^ (approximately 81%) and comparable to commercial Sr_2_Si_5_N_8_:Eu^2+^ (approximately 85%),^42–44^ and is much higher than that without Bi^3+^ doping (70.1%). This thermal stability enhancement is different from the traditional reduction of cross relaxation or thermal ionization,^45,46^ mainly ascribing to the appropriate amount of defects created by Bi^3+^ doping.

**Fig. 9:**
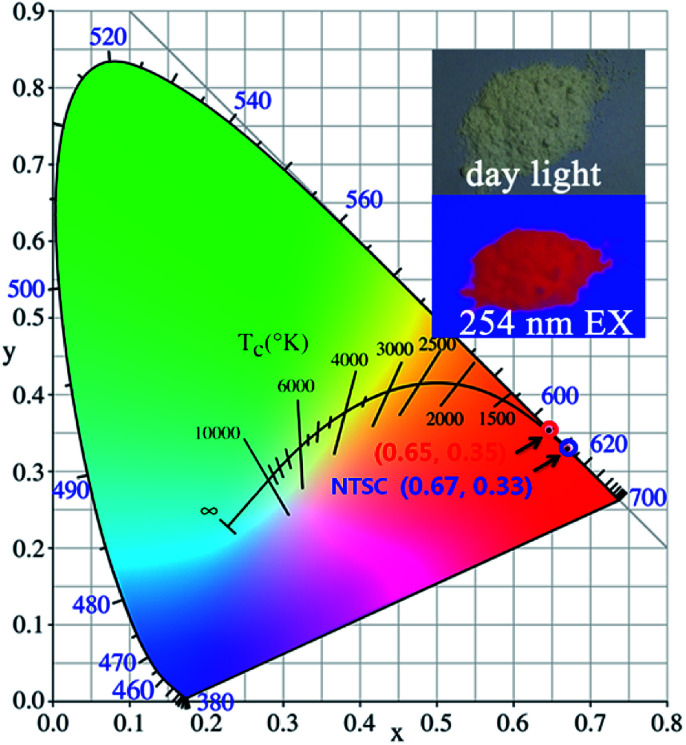
CIE coordinates of (Ca_2.83−*x*_Eu_0.17_Bi_*x*_)ZrSi_2_O_9_ (*x* = 0–0.16) phosphors under excitation at 300 nm. A digital photo of the (Ca_2.83−*x*_Eu_0.17_Bi_*x*_)ZrSi_2_O_9_ (*x* = 0.08) phosphor under daylight and a 254 nm UV lamp is shown at the upper right.

Furthermore, the CIE chromaticity coordinates for (Ca_2.83−*x*_Eu_0.17_Bi_*x*_)ZrSi_2_O_9_ (*x* = 0–0.16) excited at 300 nm were calculated, but the values were the same, which reflects the fact that Bi^3+^ doping did not change the occupancy of Eu^3+^ at any type of Ca^2+^ site. The CIE chromaticity coordinates were determined to be (0.65, 0.35) (depicted in Fig. 9), which are close to those of standard red (0.67, 0.33) given by the NTSC (National Television Standards Committee).^47^ Additionally, a digital photo of the (Ca_2.83−*x*_Eu_0.17_Bi_*x*_)ZrSi_2_O_9_ (*x* = 0.08) phosphor under daylight and a 254 nm UV lamp is shown in the inset of Fig. 9, demonstrating an intense red light emission from this phosphor under UV light excitation. With further synthetic process optimization, the luminescent properties of this phosphor are expected to be further promoted.

## 4. Conclusions

We have synthesized (Ca_2.83−*x*_Eu_0.17_Bi_*x*_)ZrSi_2_O_9_ (*x* = 0–0.16) phosphors by a conventional high-temperature solid-state reaction method using inexpensive raw materials. The DFT calculation demonstrates that the doped Bi^3+^ and Eu^3+^ prefer to occupy Ca^2+^ sites. The redshift of the charge transfer band (CTB) induced by Bi^3+^ doping of the Ca_3_ZrSi_2_O_9_:Eu^3+^ phosphor should be ascribed to the existence of a hybrid level constituted by the O 2p and Bi 6s states located above the valence band. The inner quantum yield of the optimized sample with *x* = 0.08 was promoted to 72.9%, due to the efficient sensitizing of Eu^3+^ with Bi^3+^. The formation of the appropriate amount of defects created by nonequivalence substitution, namely Bi^3+^ substituting Ca^2+^, is beneficial for increasing the thermal stability of Eu^3+^ in this phosphor. The optimized sample can remain at 84.2% of the initial quantum yield (at room temperature) when the temperature was raised to 150 °C. These parameters indicate that the Bi^3+^-doped Ca_3_ZrSi_2_O_9_:Eu^3+^ phosphor exhibits a particularly high quantum yield and excellent thermal stability, and can serve as red-emitting phosphors for future deep UV LEDs.

## Conflicts of interest

There are no conflicts of interest to declare.

## Supplementary Material
